# Prevalence and associated factors of malnutrition among indigenous infants in Sepang and Kuala Langat: A cross-sectional study

**DOI:** 10.51866/oa.643

**Published:** 2026-01-02

**Authors:** Sing Wei Low, Ai Theng Cheong, Irmi Zarina Ismail

**Affiliations:** 1 Department of Family Medicine, Faculty of Medicine and Health Sciences, Universiti Putra Malaysia, Serdang, Selangor, Malaysia.

**Keywords:** Indigenous people, malnutrition, Breastfeeding, Growth and development

## Abstract

**Introduction:**

Malnutrition has emerged as a national issue in Malaysia, especially among indigenous populations. The types of feeding practices affect infantile growth in early life. Detecting malnutrition early and increasing awareness of its contributing factors are critical in handling this issue. This study aimed to determine the prevalence and associated factors of malnutrition among indigenous infants.

**Methods:**

This cross-sectional study involved indigenous infants followed up in primary health clinics in Sepang and Kuala Langat, Selangor. Universal sampling was used to recruit participants, where survey interviews and health record reviews were conducted for data collection. Nutritional status was classified based on growth parameters (weight-for-age, height-for-age, BMI-for-age and weight-for-height Z scores) at 2, 4 and 6 months. Any abnormalities were considered to indicate malnutrition. Multiple logistic regression analyses were conducted to determine the associated factors of malnutrition.

**Results:**

A total of 119 infants were included in this study. Among them, 45.4%, 44.5% and 10.1% were exclusively breastfed, formula-fed and predominantly breastfed, respectively. Approximately 16.0% had malnutrition at 6 months of age. Multiple logistic regression analysis showed that the male infants had 3.69 and 7.37 times higher odds of having malnutrition and undernutrition than the female infants, respectively. The breastfed infants had 3.46 and 5.10 times higher odds of experiencing malnutrition and undernutrition than the formula-fed infants, respectively.

**Conclusion:**

Almost one-fifth of the indigenous infants had malnutrition at 6 months old. The male and breastfed indigenous infants were more at risk for malnutrition. However, these results should be further verified.

## Introduction

Indigenous people (Orang Asli) are defined as ‘populations with distinct social and cultural groups that share collective ancestral ties to the lands and natural resources where they live, occupy or from which they have been displaced’.^1^ They often receive government development in basic services and infrastructure late and face challenges in participating in economic activities, accessing justice and engaging in political activities. Although they make up less than 5% of the global population, 15% of the Orang Asli fall into the category of extreme poverty.^2^

The prevalence of malnutrition among the Orang Asli is high.^1^ Malnutrition is defined as deficiencies or excesses in nutrient intake and includes both undernutrition and overweight or obesity.^3^ According to the WHO’s statistics in 2019, 144 million children under 5 years old were stunted (too short for age); 47 million were wasted (too thin for height); and 38.3 million were overweight or obese.^4^

The first 2 years of a child’s life are recognised as the crucial period of growth and development.^5^ Proper growth monitoring in early infancy is important, as growth patterns are associated with a child’s present and future health.^6^ Feeding practices are one of the important and modifiable factors affecting malnutrition. Other contributing factors include antenatal health status, the presence of medical illness, maternal characteristics, housing environment and family financial and societal status.^6^ According to the WHO, all mothers are recommended to practise exclusive breastfeeding for their infants for 6 months and continue breastfeeding up to 2 years. Weaning should only be started after 6 months of age.^7^ The common types of infant feeding practices are classified into exclusive breastfeeding, predominant breastfeeding and formula feeding.

Previous local studies have determined the factors affecting malnutrition in older children. To the best of our knowledge, no study has yet investigated malnutrition in infants. Research into the factors affecting infants is important for early diagnosis and timely intervention. It is crucial to identify the types of feeding practices among indigenous infants from birth to 6 months of age to detect growth faltering early and determine potential causes of malnutrition. This study aimed to determine the prevalence and associated factors of malnutrition among indigenous infants. The results can provide insights into the extent of malnutrition and its contributing factors among indigenous infants, which can be incorporated into the development of strategies to address this issue.

## Methods

This cross-sectional study was conducted in primary care clinics that provide services to indigenous people of Sepang and Kuala Langat, Selangor. Indigenous infants aged 6–12 months who attended maternal and child health clinic appointments in Sepang and Kuala Langat during the study duration and fulfilled the inclusion criteria were recruited. Term Malaysian indigenous infants (born at 37 weeks of gestation and above)^8^ aged 6–12 months with available data on growth measurements at 2, 4 and 6 months of age were included. Indigenous infants with known genetic disorders, congenital anomalies or chronic illnesses or mothers who were medically unfit for breastfeeding were excluded.

The estimated sample size of 116 was calculated for hypothesis testing using logistic regression in G*Power (version 3.1.9; Heinrich Heine University, Dusseldorf, Germany). The calculation was based on the factor of greatest interest (breastfeeding), which showed a significant association with underweight, with a reference odds ratio of 0.328 from a previous study involving children aged 0–23 months. The sample size was determined with a study power of 80%, a significance level (a) of 0.05 and a confidence interval of 95%.^9^ Accounting for a non-response rate of 20%, we estimated the final total sample size as 139.

Data were collected for 10 months, from 1 July 2022 to 30 April 2023. A list of primary health clinics that provide services to the Orang Asli in the districts of Sepang and Kuala Langat was obtained from Unit Primer, Selangor State Health Office. According to the district health department registry, the total number of indigenous infants from July 2021 to October 2022 in Sepang and Kuala Langat was 217. All clinic baby books were reviewed to select potential participants according to the inclusion and exclusion criteria. A total of 67 indigenous infants were excluded from the study: 51 indigenous infants were born premature; two were born with congenital anomalies; three had chronic medical conditions; and 11 had incomplete growth measurements. Face-to-face interviews were scheduled on the same day as the infants’ health clinic follow-up appointments for 150 participants (mothers of eligible infants). Phone call reminders to attend appointments were made 1 day before each appointment date.

Reappointment for the next earliest available date was set for those who were unable to attend the appointment. Lastly, phone call interviews were arranged for 53 participants who were unable to attend their face-to-face interview appointments.

A data collection form was designed and used to collect the socio-demographic data of mothers (age, height, weight, educational level, employment status, number of children and total monthly family income), biodata of infants (sex, birthweight, main caretaker and history of acute medical illness) and types of feeding practices. Data were obtained from mothers during face-to-face interviews. Data regarding infant growth anthropometry measurements from birth to 6 months old were retrieved from the infant health record books. Questions regarding the types of feeding practices were adapted from the National Health and Morbidity Survey Report 2016 Volume 1 Module CH: Children Under Age 5.^10^

### Statistical analysis

Growth status was classified using the four indices of growth measurement: weight-for-age Z score, height-for-age Z score, BMI-for-age Z score and weight-for-height Z score. A weight-for- age Z score of less than –2 standard deviation (SD) was classified as underweight; more than +2 SD, high; and in between, normal. A height-for-age Z score of less than –2 SD was classified as stunting; more than +2 SD, high; and in between, normal. A BMI-for-age Z score of less than –2 SD was classified as thinness; more than +2 SD, overweight; and in between, normal. A weight- for-height Z score of less than –2 SD was classified as wasting; more than +2 SD, high; and in between, normal.

The dependent variable in this study was malnutrition, which was defined as any abnormality in the growth parameters. Maternal socio-demographic factors (age, employment status, educational level, total number of children and total family income), infant biodata (sex, birthweight, main caretaker and history of acute illness) and types of infant feeding practices (exclusive breastfeeding, predominant breastfeeding and formula feeding) were examined as the study factors.

The Statistical Package for the Social Sciences version 28.0 by IBM Corporation, Armonk, New York, United States of America was used for analysis. Continuous data were expressed as means ± SDs for normal distribution or medians and interquartile ranges (IQRs) for non-normal distribution. Categorical data were described as frequencies and percentages. The association between the study factors and malnutrition was assessed using simple logistic regression followed by multiple logistic regression. Independent variables with P<0.25 in the simple logistic regression and the types of feeding practices were included in the regression model.^11^ Multicollinearity of the independent variables was tested by examining the variance inflation factor (VIF) and tolerance statistics. VIF values below 10 and tolerance values above 0.2 indicated no collinearity within the data.^12^ Model fitness was assessed using the Hosmer—Lemeshow goodness-of-fit test. P-values of more than 0.05 indicated an adequate model fit.

### Ethical approval

This study was approved by the Medical Research and Ethics Committee of the Ministry of Health, Malaysia (NMRR ID-22-00559-DHS).

## Results

Appointments for the face-to-face and phone call interviews were set for a total of150 indigenous infants, who fulfilled the inclusion criteria to participate in the study. The total number of responses collected was 119. There were 18 mothers who did not pick up the calls; 10 remained uncontactable; and three refused to participate in the study. The response rate was 79.3% (119/150).

### Maternal socio-demographic characteristics and infant feeding profiles

Most of the mothers were young, with 64.7% aged 20–30 years. Approximately 59.7% had a total of two to four children at home. The majority of the mothers (84.9%) were unemployed, and 93.3% had a total monthly family income of less than RM 2500. Almost all mothers received formal education, while only 5.9% received no formal education. The median birthweight of the indigenous infants was 2.87 kg (IQR=0.5); the lowest birthweight was 1.8 kg, while the highest birthweight was 4.6 kg. The sex distribution of the infants in this study was fairly equal. The majority of the main caretakers were mothers (94.1%), and only 16.8% of the infants had a history of acute medical illness (Table 1).

**Table 1:** Maternal socio-demographic characteristics, infant profiles and types of feeding practices.

Variable	Frequency (N=119)	Percentage
Maternal socio-demographic characteristics		
**Age, year**		
<20	10	8.4
20-30	77	64.7
>30-40	21	17.6
>40	11	9.2
**Employment status**		
Employed	18	15.1
Unemployed	101	84.9
**Educational level**		
Primary education	38	31.9
Secondary education	71	59.7
Tertiary education	3	2.5
No formal education	7	5.9
**Number of children**		
1	39	32.8
2-4	71	59.7
≥5	9	7.6
**Monthly family income, RM**		
<2500	111	93.3
≥2500	8	6.7
**Infant profiles**		
**Sex**		
Mae	56	47.1
Female	63	52.9
**Main caretaker**		
Mother	112	94.1
Others	7	5.9
**History of acute illness**		
Yes	20	16.8
No	99	83.2
**Types of feeding practices**		
Exclusive breastfeeding	54	45.4
Predominant breastfeeding	12	10.1
Formula feeding	53	44.5

Approximately 45.4% (54/119), 10.1% (12/119) and 44.5% (53/119) of the infants were exclusively breastfed, predominantly breastfed and formula-fed, respectively.

### Malnutrition among the indigenous infants at 6 months of age

Among the indigenous infants, 16% (19/119) had malnutrition. Approximately 9.2% (11/119) were underweight at 6 months of age; 6.7% (8/119) were stunted; 2.5% (3/119) were overweight; and 5% (6/119) were wasted. Of the 19 indigenous infants with malnutrition at 6 months old, 58% (11/19) had malnutrition since 2 months of age.

About 6% (7/119), 5% (6/119) and 5% (6/119) of the infants had one, two and three abnormal growth parameters, respectively (Table 2).

**Table 2:** Malnutrition among the indigenous infants at 6 months of age.

Abnormal growth parameter	Frequency (n)	Percentage^#^
**One parameter**		
Underweight	3	
Stunting	4	3
Wasting	0	3
Overweight	0	
Sub-total	7	6
**Two parameters**		
Wasting + thinness	1	1
Underweight + stunting	3	3
Overweight + high weight-for-height Z score^*^	2	2
**Sub-total**	6	5
**Three parameters**		
Underweight + thinness + wasting	5	4
Stunting + overweight + high weight-for-height Z score^*^	1	1
**Sub-total**	6	5
**Total overall malnutrition**	19	16

* Weight-for-height Z score > +2 standard deviation

# Denominator N=119

### Factors associated with malnutrition and undernutrition among the indigenous infants at 6 months of age

Two models were analysed: the first for malnutrition and the second for undernutrition, as there were only three overweight infants. The multiple logistic regression analysis showed that the significant factors associated with malnutrition and undernutrition among the indigenous infants at 6 months of age were sex and the types of feeding practices. The male infants had 3.69 and 7.37 times higher odds of having malnutrition (Adjusted Odd Ratio (AOR)=3.69, 95% Confidence Interval (CI)=1.14, 11.99, P=0.03) and undernutrition (AOR=7.37, 95% CI=1.93, 28.06, P=0.003) than the female infants, respectively. The breastfed infants had 3.46 and 5.10 times higher odds of experiencing malnutrition (AOR=3.46, 95% CI=1.01, 11.89, P=0.049) and undernutrition (AOR=5.10, 95% CI=1.32, 19.66, P=0.018) than the formula-fed infants, respectively. The other maternal socio-demographic characteristics and infant profiles did not show a significant association with malnutrition and undernutrition in the multiple logistic regression model (Table 3).

**Table 3:** Factors associated with malnutrition and undernutrition at 6 months of age.

Variable	Model 1: Analyses for malnutrition				Model 2: Analyses for undernutrition			
	Simple logistic regression		Multiple logistic regression		Simple logistic regression		Multiple logistic regression	
	COR (95% CI)	P-value	AOR (95% CI)	P-value	COR (95% CI)	P-value	AOR (95% CI)	P-value
**Maternal characteristics**								
Age, year <30 >30	1 0.46 (0.12-1.7)	0.243	1 0.57 (0.14-2.32)	0.435	1 1.85 (0.50-6.93)	0.359		
**Employment status** Employed Unemployed	1 1.62 (0.34-7.7)	0.545			1 0.72 (0.15-3.44)	0.677		
**Educational level**								
Lower Higher	1 1.05 (0.38-2.9)	0.924			1 0.88 (0.30-2.57)	0.817		
**Number of children**								
1 >2	1 0.62 (0.23-1.69)	0.347			1 1.14 (0.39-3.35)	0.811		
**Monthly family income, RM**								
<2500 ≥2500	1 0.00 (0.00)	0.999			1 0.00 (0.00)	0.999		
**Pre-pregnancy BMI**								
Normal Abnormal	1 0.30 (0.11-0.86)	0.025	1 0.48 (0.15-1.50)	0.206	1 4.15 (1.41-12.21)	0.010	1 2.54 (0.78-8.21)	0.120
**Height**								
Stunted Not stunted	1 0.56 (0.06-5.66)	0.620			1 0.00 (0.00)	0.999		
**Infant profiles**								
**Sex**								
Female Male	1 3.87 (1.29-11.57)	0.016	1 3.69 (1.14-11.99)	**0.030**	1 6.67 (1.80-24.65)	0.004	1 7.37 (1.93-28.06)	**0.003**
**Birthweight**								
Low Not low	1 0.46 (0.13-1.65)	0.235	1 0.44 (0.11-1.80)	0.251	1 1.61 (0.40-6.42)	0.502		
**Main caretaker**								
Mother Others	1 0.00 (0.00)	0.999			1 0.00 (0.00)	0.999		
**History of acute illness**								
Yes No	1 1.87 (0.4-8.8)	0.431			1 0.62 (0.13-2.96)	0.551		
**Nutritional factor**								
**Type of feeding practices**								
Formula feeding Breastfeeding	1 3.6 (1.12-11.62)	0.032	1 3.46 (1.01-11.89)	**0.049**	1 4.49 (1.22-16.56)	0.024	1 5.10 (1.32-19.66)	**0.018**

All assumptions were fulfilled in this study before running binary logistic regression. The dependent variables were categorised as dichotomous; the observations were independent of each other; the calculated sample size was met; and there was no multicollinearity among the independent variables. Multicollinearity was checked using tolerance (>0.2) and variance inflation factor values (<5). The P-value in the Hosmer—Lemeshow test was above 0.05 (P=0.734), indicating a good fit. The model’s ability to correctly classify more than 70% of the participants indicated a good fit.

COR = Crude Odd Ratio, AOR = Adjusted Odd Ratio, CI = Confidence Interval, Significant P value < 0.05

## Discussion

This study found that 45.4% of the indigenous infants were exclusively breastfed for the first 6 months of life, which is similar to the reported 46.0% in the 2016 NHMS Report for Malaysian children under 5 years old.^13^ In Malaysia, limited studies have evaluated breastfeeding practices among indigenous people. A study of Penan infants in rural Sarawak reported that the prevalence of exclusive breastfeeding for infants under 6 months old was 44.4%.^14^ Another local study in Sepang and Carey Island, Selangor, investigated breastfeeding practices and the nutritional status of Orang Asli children. However, this study did not report the prevalence of exclusive breastfeeding. The authors found that 98.9% of Orang Asli mothers were ever breastfeeding; 33% were breastfeeding their infants for 6 months or less, and the mean time of stopping breastfeeding was 12 months of age.^15^

The prevalence of malnutrition in our study among the 6-month-old infants was 9.2% for underweight and 6.7% for stunting. These rates are lower than those reported among older indigenous children: 49% for underweight and 64% for stunting at 3–59 months of age in Krau Wildlife Reserve, Malaysia,^16^ 45.3% for underweight and 76.2% for stunting among aboriginal preschoolers in Gua Musang, Kelantan,^17^ and 35.6% for stunting among Orang Asli preschoolers in Negeri Sembilan.^18^ The prevalence of malnutrition in this study is lower because chronic malnutrition indices such as stunting developed after a prolonged period of nutrient deficiency. The prevalence of malnutrition is expected to be higher if older children are included in this population. Thus, it is important to initiate appropriate treatment measures to prevent further increments of malnutrition later.

The multiple logistic regression showed that sex and feeding practices were associated with malnutrition. There were only three overweight infants in our study; thus, the results primarily represent the undernutrition group. The male infants had 3.69 and 7.37 times higher odds of having malnutrition and undernutrition than the female infants, respectively. No similar study about indigenous infants below 6 months old was found for comparison. The 2016 NHMS study about Malaysian infants under 6 months old reported no significant association between sex and underweight.^19^ The biological factors of underlying male vulnerability are yet to be elucidated.

In their review article, Tottman et al. suggested that boys may be more responsive to growth-promoting influences and therefore more susceptible to inadequate nutrient supply. They require higher nutritional intake than girls to support optimal linear growth and body composition.^20^

In the present study, the breastfed infants were 3.46 and 5.10 times more likely to have malnutrition and undernutrition than the formula-fed infants, respectively. Although mothers’ own milk is the best source of nutrition nearly for all infants, breastfeeding may not always be possible, suitable or solely adequate for all. The quality of breastmilk could be influenced by other factors. According to the Havard Medical School in Boston, USA, the composition of human breastmilk varies with the stages of nursing, maternal diet, maternal health and environmental exposure.^21^ The protein content and fat concentration of breastmilk are dose-dependently associated with maternal diet.^21^ Additionally, mothers who exclusively breastfeed need to consume adequate amounts of vitamin D to ensure their breastmilk contains sufficient vitamin D for their infants, helping to prevent vitamin D deficiency and inadequate bone mineralisation.^22^ Nevertheless, our study did not examine in detail maternal health, diet and nursing practices that might influence the nutritional content of breastmilk. These factors should be investigated in future studies.

### Strengths and limitations

To the best of our knowledge, this study is the first to evaluate the malnutrition status in indigenous infants from birth to 6 months of age in Malaysia. The study examined anthropometric growth parameters in indigenous infants (weight for age, height for age, BMI for age and weight for height) to determine the malnutrition status and factors contributing to malnutrition.

There are a few limitations of the study that need to be discussed. The data on the types of feeding practices in this study were based on the participants’ recall of milk and fluids given from birth to 6 months of age. This method may be subject to a certain degree of recall bias, potentially leading to under- or overreporting. Further, the findings of this study cannot be generalised to the whole Malaysian indigenous population. Other factors such as maternal health and nutritional status, infant nursing practices, birth spacing and antenatal care may influence the quality of breastmilk and growth of indigenous infants. However, these factors were not examined in the current study. There were 66 participants recruited via face-to-face interviews and 53 participants interviewed via phone calls. The use of different data collection methods may have influenced the quality of the data and results.

## Conclusion

The prevalence of malnutrition is 16.0% among indigenous infants at 6 months of age. Male and breastfeeding indigenous infants are at a higher risk for malnutrition and undernutrition. However, these results should be verified in future studies in view of the limitations of this study, which did not capture some other important factors such as maternal health and nutritional status, infant nursing practices, birth spacing and antenatal care.

## Data sharing statement

Further information on the data can be requested via email from the corresponding author

**How does this paper make a difference in general practice?**

- The prevalence of malnutrition among the indigenous infants at 6 months of age in urbanised areas in this study was 16%.
- The majority of the malnourished infants at 6 months of age had malnutrition since 2 months of age (58%). Intervention is warranted as early as possible.
- The breastfed indigenous infants were at risk for undernutrition. This might be related to maternal nutritional factors, which require further investigation in future studies.
